# Dataset of running kinematics, kinetics and muscle activation at different speeds, surface gradients, cadences and with forward trunk lean

**DOI:** 10.1016/j.dib.2024.110312

**Published:** 2024-03-11

**Authors:** Bas Van Hooren, Kenneth Meijer

**Affiliations:** NUTRIM School of Nutrition and Translational Research in Metabolism, Maastricht University Medical Centre+, Department of Nutrition and Movement Sciences, Maastricht, the Netherlands

**Keywords:** Biomechanics, Kinematics, Kinetics, Muscle activation, EMG, OpenSim, C3D files, Muscle force

## Abstract

**Data collection process:**

This dataset includes running biomechanics measured using an instrumented treadmill combined with three- dimensional motion capture and surface muscle activation among 19 healthy participants (10 males, 9 females, mean ± SD age 23.6 ± 3.7 years, body height 174.9 ± 9.2 m; body mass 67.2 ± 10.4 kg) of various performance levels (untrained to Olympic level athlete). Running biomechanics were measured during thirteen different treadmill running conditions that included five speeds (2.78, 3.0, 3.33, 4.0 and 5.0 m·s^−1^), four gradients (-6, -3, +3, +6°), three cadences (preferred, ± 10 steps·min^−1^), and a condition with forward trunk lean.

**Dataset:**

Both raw data (.C3D files that include the 3D positions of the markers, ground reaction forces, and surface muscle activation) and processed data (joint angles, joint moments, muscle forces, joint contact forces, all analysed using OpenSim 3.3 musculoskeletal modelling software) are available. Standard subject characteristics such as height and body mass are also available.

**Reuse potential:**

The data can be re-used for various purposes including model validation studies, studies that aim to investigate biomechanical outcomes in specific running conditions, and as a reference database of running biomechanics across various conditions in healthy young non-injured individuals.

Specifications Table

**Table:** 

Subject	Sport Sciences, Therapy and Medicine
Specific subject area	Biomechanics of treadmill running in various conditions
Type of data	C3D files with unfiltered marker coordinates, unfiltered ground reaction forces and center of pressure, and unfiltered surface muscle activation for eleven muscles. .trc and .mot files with marker coordinates and ground reaction forces suitable for OpenSim 3.3. These files are provided for a static trial and for all running trials. The dataset also contains scaled OpenSim models, and the output from inverse kinematics, inverse dynamics, dynamic optimization, and joint reaction analysis. MATLAB and Excel files with additional outcomes computed from the OpenSim files are also available.
Data collection	The computer assisted rehabilitation environment (CAREN, Motek, The Netherlands) system combines an instrumented split-belt treadmill (ForceLink, Culemborg, The Netherlands, individual belt length and width 2.15 × 0.5 m, 6.28-kW motor per belt, 60 Hz belt speed update frequency, and 0-18 km·h^−1^ speed range, 1000 Hz) with a 12-camera three- dimensional motion capture system (VICON NEXUS v2.1, Oxford Metrics Group, Oxford, UK, 100 Hz) and was used to measure kinetic and kinematic outcomes. Surface muscle activation was measured in eleven muscles using parallel-bar dry surface electrodes (Trigno™ Wireless System, DelSys, Inc, Boston, MA, USA, 27 × 37 × 13 mm, 14 gram, inter-electrode distance 10 mm). The data were further processed using OpenSim 3.3 with the Catelli model. The OpenSim output was used to compute the load at specific tissues (patellofemoral joint, tibia, Achilles tendon) as detailed in [1]. All data is available both in non-normalized and time-normalized format.
Data source location	Institution: Maastricht University Medical Centre+ City/Town/Region: Maastricht Country: Netherlands
Data accessibility	Repository name: OpenScience Framework Data identification number: DOI 10.17605/OSF.IO/7QBXC Direct URL to data: https://osf.io/7qbxc/ Instructions for accessing these data: All data is organized in folders with intuitive naming and can be downloaded without restrictions.
Related research article	Van Hooren, B.; van Rengs, L.; Meijer, K.; Per-step and cumulative load at three common running injury locations: the effect of speed, surface gradient and cadence. Scandinavian Journal of Medicine and Science in Sports. 2024. 34(2); e14570

## 1 Value of the Data

• The data can be used for modelling studies on running biomechanics (e.g., validating new models or new modelling procedures by comparing modelled muscle forces against measured surface muscle activation);
• The data can improve understanding of biomechanical differences between running at different speeds, gradients, and step frequencies;
• The data can be used as part of a reference database for running biomechanics in healthy non-injured individuals;
• The range in speeds and training level ensure the data can be used to reflect different young and healthy populations;
• The data can be useful for any scientist interested in running biomechanics;
• The data can be used for any scientific purposes in case permission is granted

## 2 Background

The original motivation for collecting this data was two: 1) quantify tissue loading during various running conditions with the aim to inform on appropriate loading for injury prevention and rehabilitation, 2) use machine learning models to predict the tissue load at various running conditions using wearable sensors.

## 3 Data Description

All data can be found at the OpenScience framework. The OSF storage folder contains the subfolders described in Table 1, which contain all data. The folder also contains an Excel file with basic subject characteristics, and .xml files with the settings used for scaling, inverse kinematics and inverse dynamics procedures. Each file starts with a subject number, followed by an underscore and an abbreviation of the condition. For example, Sub_01_278ms refers to the 2.78 m·s^−1^ condition for subject 01.

**Table 1:** Coding and structure of the dataset.

Folder	File typ e	Content	Generated	Naming	Additional
0. C3D files	.C3D	Raw 3D marker positions, ground reaction forces and surface muscle activation for the static calibration, running trials, and for trials in which the maximum voluntary contraction has been recorded	Vicon Nexus	Subject_ number_ condition	/
1. Scaling files OpenSim	.trc, .mot	Raw 3D marker positions (.trc) and ground reaction forces (.mot) for the static calibration trial in OpenSim 3.3 file format	MATLAB	Subject_ number_ condition	/
2. Scaled OpenSim models	.osim	Scaled Catelli OpenSim model	OpenSim 3.3	Subject_ number_ condition	The folder contains both models with their original strength and models in which the maximum isometric muscle force has been increased 2 and 3 times.
3. GRF, marker, event files	. mot, .trc, .xlsx	Raw 3D marker positions (.trc) and ground reaction forces (.mot) for the running trials in OpenSim 3.3 file format. The .xlsx file contains the time points of initial contact and toe-off for each foot during the running period analyzed	MATLAB	Subject_ number_ condition	/
4. IK output	.mot	Joint angles determined with the inverse kinematics (IK) procedure	OpenSim 3.3	Subject_ number_ condition	/
5. ID output	.sto	Net joint moments determined with the inverse dynamics (ID) procedure	OpenSim 3.3	Subject_ number_ condition	/
6. DO output	.sto	Files with the muscle forces, muscle activation, (non)normalized fiber length and velocity, and reserve actuator contribution determined with dynamic optimization (DO)	MATLAB	Subject_ number_ condition	/
7. JRA output	.sto	Files with the output from joint reaction analysis expressed in different reference frames	MATLAB	Subject_ number_ condition	/
8. Tissue loading	.xlsx	Excel files with the calculated loading indices on the patellofemoral joint, tibia, and Achilles tendon	MATLAB	Subject_ number_ condition	/
9. Time normalized data	.xlsx	Excel files with time normalized data over all analyzed steps per subject. Each file contains the mean and standard deviation for the ground reaction forces, inverse kinematics, inverse dynamics, muscle forces, and tissue loads	MATLAB	Subject_ number_ condition	/
10. Non-time normalized data	.xlsx	Excel files with non-time normalized data over all analyzed steps per subject. Each file contains the mean and standard deviation for the ground reaction forces, inverse kinematics, inverse dynamics, muscle forces, and tissue loads	MATLAB	Subject_ number_ condition	/

The conditions included within the dataset are 1) level running at different speeds: 2.78, 3.00, 3.33, 4.00, 5.00 m·s^−1^, 2) running at two uphill and downhill gradients: -6, -3, and +3, +6 degrees at 2.78 m·s^−1^, and 3) running with a higher and lower step frequency compared to the preferred step frequency at 3.33 m·s^−1^. Note that we also included data from running with forward trunk lean. This data was not included in the original paper, but will be used in future papers.

Most files start from zero velocity, then increase in speed until a full minute of running at the desired speed was recorded, and then slow down to zero velocity.

**OpenSim 3.3 vs 4.x**

All data analyses was done in OpenSim version 3.3. OpenSim version 4 and later has several updates that result in some incompatibility issues. To ensure the present data can be used within OpenSim 4 and later some modifications may therefore be required. For example, while the models (.osim) and ground reaction force (.mot) can also be loaded within OpenSim 4 and later, the 'times' column header in the marker files (.trc) needs to be changed to 'time'. Further, changes to the joint definitions in the models made for OpenSim 4 and later (see also https://simtk-confluence.stanford.edu:8443/display/OpenSim/Upgrade+Notes+for+OpenSim+4.0) result in erroneous movements for some joints (e.g., patellofemoral joint) when using the inverse kinematics output from OpenSim 3.3 within OpenSim 4 and later. Users that would like to use the present results in OpenSim 4 and later may therefore want to re-do the inverse kinematics to ensure the results match with the new model joint definitions.

## 4 Experimental Design, Materials and Methods

All participants completed a single test session and were instructed to avoid strenuous activity for 36 h, alcohol for 24 h, caffeine for 6 h and a heavy meal 1 h before the session. When participants entered the lab, we took anthropometric measurements using standardized procedures. The participants were then equipped with retroreflective markers. After subject calibration and a familiarization period, the participants completed short (1 min) runs while ground reaction forces and lower body and trunk kinematics were collected.

The computer assisted rehabilitation environment (CAREN, Motek, The Netherlands) system was used to measure kinetic and kinematic outcomes and combines an instrumented split-belt treadmill (belt length and width 2.15 × 0.5 m, 6.28-kW motor per belt, 60 Hz belt speed update frequency and 0-5 m·s^−1^ speed range) with a 12-camera three-dimensional motion capture system (VICON NEXUS v2.1, Oxford Metrics Group, Oxford, UK, 100 Hz). Each participant ran in their own shoes to maximize the applicability of the findings to in-field conditions.

After calibration of the systems, 26 retroreflective skin markers with a diameter of 14 mm were attached to the skin with double-sided tape using the using a modified lower-limb and trunk marker (Human Body Model v2) [2].

Prior to data collection, the participants were instructed to run for 8 min at a fixed-paced speed of 2.78 m·s^−1^ to familiarize themselves with treadmill running [3]. No additional familiarization was performed prior to each individual condition. This was followed by 4 minutes of running at 3.33 m·s^−1^. The participants then completed a series of 1-minute runs at different fixed-paced speeds and treadmill slopes, with the order of conditions being randomized by an online research randomizer (https://www.randomizer.org/). These speeds and slopes were selected to reflect conditions that recreational runners could encounter when running in-field. After these conditions, the participants ran at 3.33 m·s^−1^ with a higher and lower step frequency (± 10 steps·min^−1^) compared to their self-selected step frequency during the 4 min trial at 3.33 m·s^−1^. The lower and higher cadence was enforced using a metronome. Note that we also included data from running with forward trunk lean. This data was not included in the original paper, but will be used in future papers. Forward trunk lean was achieved by instructing participants to run with a slight forward trunk lean and the degree to which this was successfully achieved was monitored visually. The order of the cadence and trunk lean conditions was also randomized. The participants were allowed to take rest periods between trials when required and were instructed to run as if they were running outside and focus on the simulated virtual environment.

Raw marker data were labelled in Vicon Nexus, gaps were filled with a combination of pattern, spline, and rigid body fills and smoothed with a Woltring quintic spline filter [4]. The processed kinematic and kinetic data were then exported to musculoskeletal simulation software (OpenSim SimTK 3.3, Stanford, USA) [5] using custom-made MATLAB scripts. Musculoskeletal simulation was performed using a modified full-body musculoskeletal model (22 rigid body segments, 37 degrees of freedom and 80 muscles) that was designed to produce more realistic moment arms during tasks involving large hip and knee flexions [6]. Joint angles were computed using a weighted least squares minimization of markers on segments and joints to match the virtual markers on the scaled model to the experimental markers during each frame using the “Inverse kinematics” option, while net joint moments for each degree of freedom were computed using the “Inverse dynamics” option. A dynamic optimization criterion as described by De Groote [7] was used to determine the muscle forces required to reproduce the joint moments. This optimization used a cost function that minimized the sum of the squared muscle activations and accounted for the force-length-velocity relationship of muscles, and also accounted for tendon compliance in contrast to the commonly used static optimization. The “Joint Reaction analysis” tool [8] was used to calculate patellofemoral and ankle contact forces by vector summing the joint reaction/intersegmental forces from inverse dynamics and muscle forces from dynamic optimization. More details on the experimental design and data analysis can be found in [1].

## Limitations

Two participants had small changes only in step frequency during the trials in which step frequency was manipulated. This is also described in the original article. For one subject, the forward trunk lean trial is missing.

## Ethics Statement

The study was approved by the local ethics committee (nr. 2019-1138) was conducted according to the declaration of Helsinki and all participants signed informed consent form prior to the measurements.

## CRediT authorship contribution statement

**Bas Van Hooren:** Conceptualization, Methodology, Investigation, Formal analysis, Writing – original draft. **Kenneth Meijer:** Writing – review & editing, Supervision.

## Data Availability

Dataset of running biomechanics across different speeds, gradients and cadences (Original data) (OpenScience Framework).

## References

[1] Van Hooren B., Van Rengs L., Meijer K. (2024). Per-step and cumulative load at three common running injury locations: the effect of speed, surface gradient and cadence. Scand. J. Med. Sci. Sports.

[2] van den Bogert A.J., Geijtenbeek T., Even-Zohar O., Steenbrink F., Hardin E.C. (2013). A real-time system for biomechanical analysis of human movement and muscle function. Med. Biol. Eng. Comput..

[3] Van Hooren B., Fuller J.T., Buckley J.D. (2020). Is motorized treadmill running biomechanically comparable to overground running? A systematic review and meta-analysis of cross-over studies. Sports Med..

[4] Woltring H.J. (1986). A Fortran package for generalized, cross-validatory spline smoothing and differentiation. Adv. Eng. Software.

[5] Delp S.L., Anderson F.C., Arnold A.S. (2007). OpenSim: open-source software to create and analyze dynamic simulations of movement. IEEE Trans. Biomed. Eng..

[6] Catelli D.S., Wesseling M., Jonkers I., Lamontagne M. (2019). A musculoskeletal model customized for squatting task. Comput. Methods Biomech. Biomed. Eng..

[7] De Groote F., Kinney A.L., Rao A.V., Fregly B.J. (2016). Evaluation of direct collocation optimal control problem formulations for solving the muscle redundancy problem. Ann. Biomed. Eng..

[8] Steele K.M., Demers M.S., Schwartz M.H., Delp S.L. (2012). Compressive tibiofemoral force during crouch gait. Gait Posture.

